# ACEP's Recommendations for Brain Computed Tomography Scan in Adult Minor Head Trauma Patients; a Diagnostic Accuracy Study

**Published:** 2020-10-29

**Authors:** Mohammad Mohammaddoust, Niaz Mohammad Jafari Chokan, SeyedehMaryam MoshirianFarahi, Ayoub Tavakolian, Mahdi Foroughian

**Affiliations:** 1 Mohammad Mohammaddoust, MD: Department of Emergency Medicine, Mashhad University of Medical Sciences, Mashhad, Iran.; 2 Niaz-Mohammad Jafari Chokan, MD: Department of Emergency Medicine, Faculty of Medicine, Mashhad University of Medical Sciences, Mashhad, Iran.; 3 Seyedeh Maryam Moshirian Farahi: Department of Psychology, Salman Institute of Higher Education, Mashhad, Iran.; 4 Ayoub Tavakolian, MD: Department of Emergency Medicine, Faculty of Medicine, Sabzevar University of Medical Sciences, Sabzevar, Iran.; 5 Mahdi Foroughian, MD: Department of Emergency Medicine, Faculty of Medicine, Mashhad University of Medical Sciences, Mashhad, Iran.

**Keywords:** Craniocerebral Trauma, Brain, Tomography, X-Ray Computed, Health Planning Guidelines

## Abstract

**Introduction:**

Some clinical decision rules have been developed to identify minor head trauma (MHT) patients in need of brain computed tomography (CT) scan for detection of possible traumatic brain injuries (TBIs). This study aimed to evaluate the performance of American College of Emergency Physicians (ACEP) recommendations in this regard.

**Methods:**

This study is a cross-sectional study of MHT (GCS: 13-15) cases who referred to emergency department of a level one trauma center, Mashhad, Iran, from October 2017 to March 2018. The screening performance characteristics of ACEP recommendations for performing brain CT scan in these patients were calculated.

**Results:**

500 patients with a mean age of 37.97 ± 15.96 years were evaluated. Based on level one recommendations, 73 (14.6 %) patients had to be assessed by brain CT scan. 67 (91.8%) were assessed and 6 (8.2%) were not assessed based on decision of their in-charge physician. According to level two recommendations, 125 (25.0%) patients did not need brain CT scan, 85 (68%) of whom had been assessed (all normal). Performing brain CT scan according to the level one recommendation of ACEP’s clinical policy showed 29.6% sensitivity (95% CI: 13.75 to 50.18) and 86.3% specificity (95% CI: 82.68 to 89.14). The overall ACEP’s clinical policy for neuroimaging of adults with MTBI showed sensitivity and specificity of 92.59% (95% CI: ‎75.71 to 99.09) and 26.4% (95% CI: 22.51 to 30.65), respectively.

**Conclusion:**

ACEP’s clinical policy has a high-level sensitivity for using brain CT scan in detection of probable TBI in patients with MHT.

## Introduction

Minor Head Trauma (MHT) is blunt trauma to the head in patients with GCS scores between 13 to 15 secondary to the trauma (1). Traumatic brain injury (TBI) indicates an injury to the brain itself and more than 75% of treated TBI cases are mild (2). Mild TBI is a common neurological disorder and only 0.4% to 1% of these injuries require neurosurgical intervention (3). The most important issue in patients with minor head trauma is to identify patients with probable intracranial injuries who require hospitalization and proper management. Brain CT scan is the standard imaging modality for detecting intracranial injury of trauma patients in emergency department (ED). Most of these patients (80-90%) do not need to be admitted, and almost all of them are discharged with appropriate instructions. The common use of brain CT scans is associated with exposure to ionizing radiation and high healthcare costs, considering the large number of people affected (4). Therefore, some clinical decision-making rules have been developed to find MHT patients who are susceptible to intracranial lesions on CT scans in ED. Some of the important clinical decision rules are the Canadian CT Head Rule (CCHR), the New Orleans Criteria (NOC), the National Emergency X-Radiography Utilization Study II (NEXUS II) criteria, and American College of Emergency Physicians (ACEP) Clinical Policy (5, 6). In many studies, the efficacy of these guidelines, such as CCHR, NOC, and NEXUS II, has been evaluated (7-9). However, the best guideline to be used in routine practice may be highly dependent on the ED’s policies and situation and level of infrastructures availability (7-12).

According to one study, which was done in Taiwan, the sensitivity and specificity of ACEP guideline were 75.97% and 34.68%, respectively. The study concluded that, although ACEP guideline reduced unnecessary brain CT scans, it wasn't an appropriate guideline for use in Taiwan (13). Based on the above-mentioned points, this study aimed to evaluate the accuracy of ACEP's recommendation in identifying MHT patients in need of brain CT scan and ruling out of susceptible intracranial injuries.

## Methods

***Study design and setting***

This prospective cross-sectional study was conducted on adult MHT patients (GCS score of 13 to 15 one day after trauma) who referred to the ED of a hospital affiliated to Mashhad University of Medical Sciences (level one trauma center), Mashhad, Iran, from 2017 to March 2018. The screening performance characteristics of ACEP recommendations for performing brain CT scan in these patients were calculated. This study has been approved by the Ethics Committee of Mashhad University of Medical Sciences under the Ethics code of IR.MUMS.fm.REC.1396.70.

***Participants***

This study has evaluated patients with minor head trauma (GCS≥13), 18 years old or older who referred to the trauma center. Patients aged less than 18 years and those with an obvious penetrating skull injury, unstable vital signs associated with major trauma, and pregnancy were excluded.

***Study Protocol***

The ACEP recommendations were taught to emergency residents responsible for data gathering. Brain CT scans were performed based on the opinion of emergency physicians. Patients who were discharged without a brain CT scan were followed in 2 weeks and were asked to come back to the ED immediately if they encountered any unusual symptoms (as previously announced). Therefore, patients who were asymptomatic after 2 weeks were considered as brain damage-free cases. 

***Data gathering***

After history taking and performing physical examination, demographic characteristics and clinical symptoms and signs of the patients were recorded on a checklist that had been designed based on ACEP recommendations (1).

***Statistical analysis***

The data were entered into SPSS 22 (SPSS Inc., Chicago, Illinois, USA). To assess the accuracy of ACEP recommendations, sensitivity, specificity, positive and negative predictive values, and positive and negative likelihood ratios as well as area under the receiver operating characteristic (ROC) curve were calculated and reported with 95% confidence interval (CI). The findings were presented as mean ± standard deviation or frequency (%).

## Results

***Baseline characteristics of studied cases***

500 patients with a mean age of 37.97 ± 15.96 (18 – 90) years were evaluated (67% male). Table 1 shows the baseline characteristics of studied case. The most frequent trauma mechanisms were motor vehicle collision (34.8%) and direct hitting on the head (34.4%), respectively. Brain CT scan was done for 404 (80.0%) cases based on the in-charge physicians’ decision.

The frequency of ACEP-recommended variables for doing brain CT scan have been shown in table 2. The most frequent symptoms that indicated performance of brain CT scan based on level one and two recommendations, were headache (42.2%) and dangerous mechanism of trauma (39%), respectively. 

***Brain CT scan; Indications and Findings***

***Level one recommendation***

Based on level one recommendations, 427 (85.4%) patients did not need brain CT scan, 337 (78.9%) of which were assessed by CT scan, based on the decision of their in-charge physician (318 (94.4%) normal, 2 (0.59%) intracranial hemorrhage, 1 (0.29%) epidural hematoma, 4 (1.18%) subarachnoid hemorrhage, and 12 (3.56%) skull fracture).

Based on this level of recommendation, 73 (14.6 %) patients should have been assessed by brain CT scan [6 (8.2%) were not assessed based on decision of their in-charge physician (1(1.5%) intracranial hemorrhage, 1 (1.5%) epidural hematoma, 1(1.5%) subarachnoid hemorrhage, 1 (1.5%), intra-ventricular hemorrhage and 2 (6%) skull fracture]. 

***Level two recommendation***

Based on level two recommendations, 125 (25.0%) patients did not need brain CT scan, 85 (68%) of which had been assessed (all normal). Also based on this level of recommendations, 375 (75.0%) patients should have been assessed by CT scan, 319 (85%) of which had been assessed (292 (91.5%) normal, 3(0.94%) intracranial hemorrhage, 2 (0.65%) epidural hematoma, 5 (1.6%) subarachnoid hemorrhage, 1 (0.31%) intra-ventricular hemorrhage, and 16 (5%) skull fracture).

***Screening performance characteristics of ACEP recommendations***

Doing brain CT scan according to level one recommendations of ACEP’s clinical policy showed 29.6% sensitivity (95% CI: 13.75 to 50.18) and 86.3% specificity (95% CI: 82.68 to 89.14). Overall, ACEP’s clinical policy for neuroimaging of adults with MTBI showed sensitivity and specificity of 92.6% (95% CI: ‎75.71 to 99.09) and 26.4% (95% CI: 22.51 to 30.65), respectively (table 3). 

**Table 1:** Baseline characteristics of study population

**Characteristics**	**Number (%)**
**Gender**	
Male	335 (67.0)
Female	165 (33.0)
**Age (year)**	
≤ 60	449 (89.8)
> 60	51 (10.2)
**Trauma mechanism**	
Falling down	30 (6.0)
Motor vehicle collision	174 (34.8)
Passenger-related accident	60 (12.0)
Hitting injury (assault)	172 (34.4)
Other	64 (12.8)
**Signs and symptoms**	
Post-traumatic amnesia	54 (10.8)
Vomiting	37 (7.4)
Short memory loss	22 (4.4)
Coagulopathy	3 (0.6)
Decreased level of consciousness	31 (6.2)
Moderate headache	88 (17.6)
Severe headache	123 (24.6)
Intoxication	16 (3.2)
Trauma above the clavicle	115 (23.0)

**Table 2:** Frequency of level one and two ACEP-recommended variables in the study population

**Variables**	**Number (%)**
**Level one**	
Post-traumatic amnesia	54 (10.8)
Vomiting	37 (7.4)
Short memory loss	22 (4.4)
Coagulopathy	3 (0.6)
Headache	211 (42.2)
Age> 60 year	51 (10.2)
Intoxication	16 (3.2)
Trauma above the clavicle	115 (23.0)
Post-traumatic seizure	0 (0.0)
Focal neurological deficit	0 (0.0)
**Level two**	
Focal neurological deficit	0 (0.0)
Vomiting	37 (7.4)
Severe headache	123 (24.6)
Age ≥ 65 year	40 (0.8)
Basilar skull fracture	0 (0.0)
GCS < 15	31 (6.2)
Coagulopathy	3 (0.6)
Dangerous mechanism of injury	195 (39.0)

**Table 3:** Screening performance characteristics of ACEP-recommended criteria (level one, two, and overall) for performing brain computed tomography scan in patients with minor head trauma

**Characters**	**Level one ** **‎**		**Level two**		**One + two (0verall)**	
	**Value**	**95% CI**	**Value**	**95% CI**	**Value**	**95% CI**
Sensitivity	29.61	13.75 - 50.18	89.49	66.86 -98.70	92.59	75.71- 99.09
Specificity	86.28	82.68- 89.14	26.33	21.95- 31.95	26.41	22.51- 30.65
PPV	11.00	6.76 - 20.24	6.80	5.81- 7.94	7.80	7.06 - 8.80
NPV	94.00	92.88 - 95.55	97.70	91.88- 99.38	97.00	91.70 - 99.39
PLR	2.12	1.15 - 3.99	1.22	1.03 -1.44	1.30	1.12 - 1.42
NLR	0.81	0.64 - 1.05	0.39	0.10 - 1.48	0.32	0.07 - 1.07
Accuracy	80.59	76.50 - 84.43	30.21	25.41- 35.48	27.21	22.94 - 31.85

CI: Confidence interval; PPV: Positive predictive value; NPV: Negative predictive value; PLR: Positive likelihood ratio; NLR: Negative Likelihood Ratio.

## Discussion

This study, found that the ACEP criteria for conducting brain CT scan in traumatic brain injury is highly sensitive (92.59%) for finding pathologies in patients with minor head trauma.

The ACEP criteria identify patients who need to undergo a brain CT scan in two levels (14). Based on the criteria, patients who have symptoms or signs according to the first level of recommendation should be assessed via CT scan (14). This study shows that conducting a brain CT scan only based on the first level of recommendation has low sensitivity (29.6%) but relatively high specificity (86.3%). The high specificity of the first level of this criteria shows that the recommendation of ACEP criteria for conducting brain CT scan for all patients corresponding to the first level of ACEP criteria is completely reasonable (14), while low sensitivity of the first level of recommendation shows that conducting brain CT scan only based on the first level of recommendation leads to missing many patients with TBI (15). The second level of ACEP criteria states that CT scan should be performed only for some of the patients corresponding to this level (16). The results of our study confirm the recommendation of the ACEP criteria. When patients corresponding to the second level of recommendations underwent a CT, the sensitivity of this study extremely increased, while it's specificity extremely decreased, which means that the number of patients undergoing CT scan with no indication and normal CT results increased. Therefore, based on these criteria it's better to observe patients corresponding to the second level of ACEP criteria and not conduct a brain CT scan once they arrived at the emergency department. This observation will indicate which patients need to undergo brain CT scan (17).

The sensitivity of our study was higher than the study performed in Taiwan, while the specificity of our study was lower (13). The review of literature shows that similar studies, which investigate the effectiveness of the ACEP criteria, are rare. While several studies have investigated other guidelines such as CCHR, NOC, and NEXUS II. In these studies, the sensitivities of CCHR and NOC were (100, and 95 %) and (100-99%), respectively; also, the specificities of CCHR and NOC were (47-70%) and (3-31%), respectively (1, 7, 13, 18-20). Another study, which has been done on the NEXUS II criteria, has reported the sensitivity and specificity of this guideline as (100%) and (33%), respectively (21). Ro and et al. compared the predictive performance of CCHR, NOC, and NEXUS II, for detecting clinically important TBI. The mentioned study showed that their sensitivity and specificity for clinically important brain injury were as follows: CCHR, (79.2%, 95%) and (41.3%, 95%); NOC, (91.9%, 95%) and (22.4%, 95%); and NEXUS-II, (88.7%, 95%) and (46.5%, 95%), respectively (12).

In the present study, the sensitivity found for ACEP was similar to those found for other guidelines in previous studies, and it was shown that it can detect almost all pathological cases of mild TBI; whereas the specificity of ACEP is lower than CCHR and NEXUS II and higher than NOC. As a result, compared to other guidelines, CCHR could best decrease excessive use of CT. Though ACEP guidelines increased using CT scan, if physicians pay further attention to choose patients in the second level of the recommendation of ACEP to do a CT scan it will be a useful guideline.

## Limitations

This study only assessed the ACEP guideline and did not assess other guidelines to compare them. The study was done in a single trauma center. Some patients cooperated poorly and were, therefore, excluded from this study.

## Conclusion:

The findings revealed that ACEP's recommendation for performing brain CT scan in MHT patients has 92% sensitivity and 26% specificity. It could be a useful guideline to decrease the number of unnecessary CT scans, reduce radiation, and avoid extra costs for the patient and the healthcare system.
